# Burrow ambient temperature influences *Helice* crab activity and availability for migratory Red‐crowned cranes *Grus japonensis*

**DOI:** 10.1002/ece3.6788

**Published:** 2020-09-18

**Authors:** Donglai Li, Jing Zhang, Lingyu Chen, Huw Lloyd, Zhengwang Zhang

**Affiliations:** ^1^ Provincial Key Laboratory of Animal Resource and Epidemic Disease Prevention College of Life Sciences Liaoning University Shenyang China; ^2^ Ministry of Education Key Laboratory for Biodiversity Science and Ecological Engineering College of Life Sciences Beijing Normal University Beijing China; ^3^ Ecology and Environment Research Centre Department of Natural Sciences Manchester Metropolitan University Manchester UK

**Keywords:** coastal wetlands, diet composition, fecal analysis, generalized linear models, *Helice tientsinensis*, migration, prey availability, Red‐crowned cranes

## Abstract

For migratory birds that specialize on particular benthic macroinvertebrate species, the timing of migration is critical since prey availability may be temporally limited and a function of local ambient temperature. Hence, variation in local ambient temperature can influence the diet composition of migrant birds, and, consequently, they may be constrained by which stopover and wintering sites they are able to utilize during periods of colder temperatures. Here, we use fecal analysis, observer‐based population counts, digital video recordings, and temperature data to test five predictions regarding the influence of local ambient temperature on the activity and availability of mudflat crabs—a key prey resource at three staging/wintering sites in eastern China, for migratory Red‐crowned cranes (*Grus japonensis*) and how this subsequently influences crane diet and use of wetland sites. Pearson's correlations and generalized linear models revealed that mudflat crabs became significantly more surface active with increasing burrow ambient temperature. Piecewise regression analysis revealed that crab surface activity was largely limited to a burrow ambient temperature threshold between 12 and 13℃ after which activity significantly increased. Crab activity declining temporally during the crane's autumn migration period but increased during spring migration. Crabs accounted for a significant proportion of crane diet at two of three sites; however, the frequency of crab remains was significantly different between sites, and between autumn and spring migration. Analyses of crane count data revealed a degree of congruence between the migration timing of Red‐crowned cranes with periods of warmer ambient temperature, and a significant, positive correlation between the percentage of crab remains in crane feces and site ambient temperature. Collectively, our data suggest that temperature‐related mudflat crab activity may provide an important time window for migratory Red‐crowned cranes to utilize critical stopover sites and the crabs’ food resources.

## Graphical abstract

Mudflat crabs became significantly more surface active with increasing burrow ambient temperature, and the crab surface activity was largely limited to a burrow ambient temperature threshold between 12 and 13°C after which activity significantly increased. Crabs accounted for a significant proportion of crane diet at two of three sites; however, the frequency of crab remains was significant different between sites, and between autumn and spring migration. A degree of congruence between the migration timing of Red‐crowned cranes with periods of warmer ambient temperature, and a significant, positive correlation between the percentage of crab remains in crane feces and site ambient temperature.

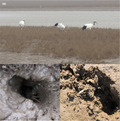

## 1 INTRODUCTION

Benthic macroinvertebrates (e.g., polychaetas, bivalves, and crustaceans) serve as critical nutrient‐rich prey resources for many migratory waterbird species at coastal saltmarsh and intertidal mudflat stopover or wintering sites (Anders, Churchyard, & Hiddink, 2009; Castro & Myers, 1993; Choi et al., 2017; Piersma, 1987; Yang et al., 2013). Some prey species, however, become less available during periods of cold weather (Esselink & Zwarts, 1989; Zwarts & Wanink, 1993) because of lower activity levels with colder temperatures, and so become harder to detect by migratory birds (Evans, 1979; Goss‐Custard, 1969; Pienkowski, 1983). A number of migratory bird species including gulls, curlews, and cranes specialize on populations of shallow‐water and intertidal crab species (Decapoda; Brachyura; Varunidae) at their stopover or wintering areas (Beron, Garcia, Luppi, & Favero, 2011; Ellis, Chen, O'Keefe, Shulmanb, & Witmana, 2005; Li, Ding, Yuan, Lloyd, & Zhang, 2014; Piersma, 1986). During periods of warmer temperatures, crabs become more active and increase their own feeding efficiency when ambient temperatures reach above a mean or maximum daily temperature threshold (Barbeau & Scheibling, 1994; Matheson & Gagnon, 2012; Rebach, 1974; Siikavuopio & James, 2015). Thus, crab prey availability may be a function of ambient temperature at stopover or wintering areas for crab specialist migratory bird species during narrow temporal windows for migration. Consequently, these bird species may be more adversely affected and more constrained by which stopover and wintering sites they are able to utilize during periods of colder, more severe winter weather.

**FIGURE 1:**
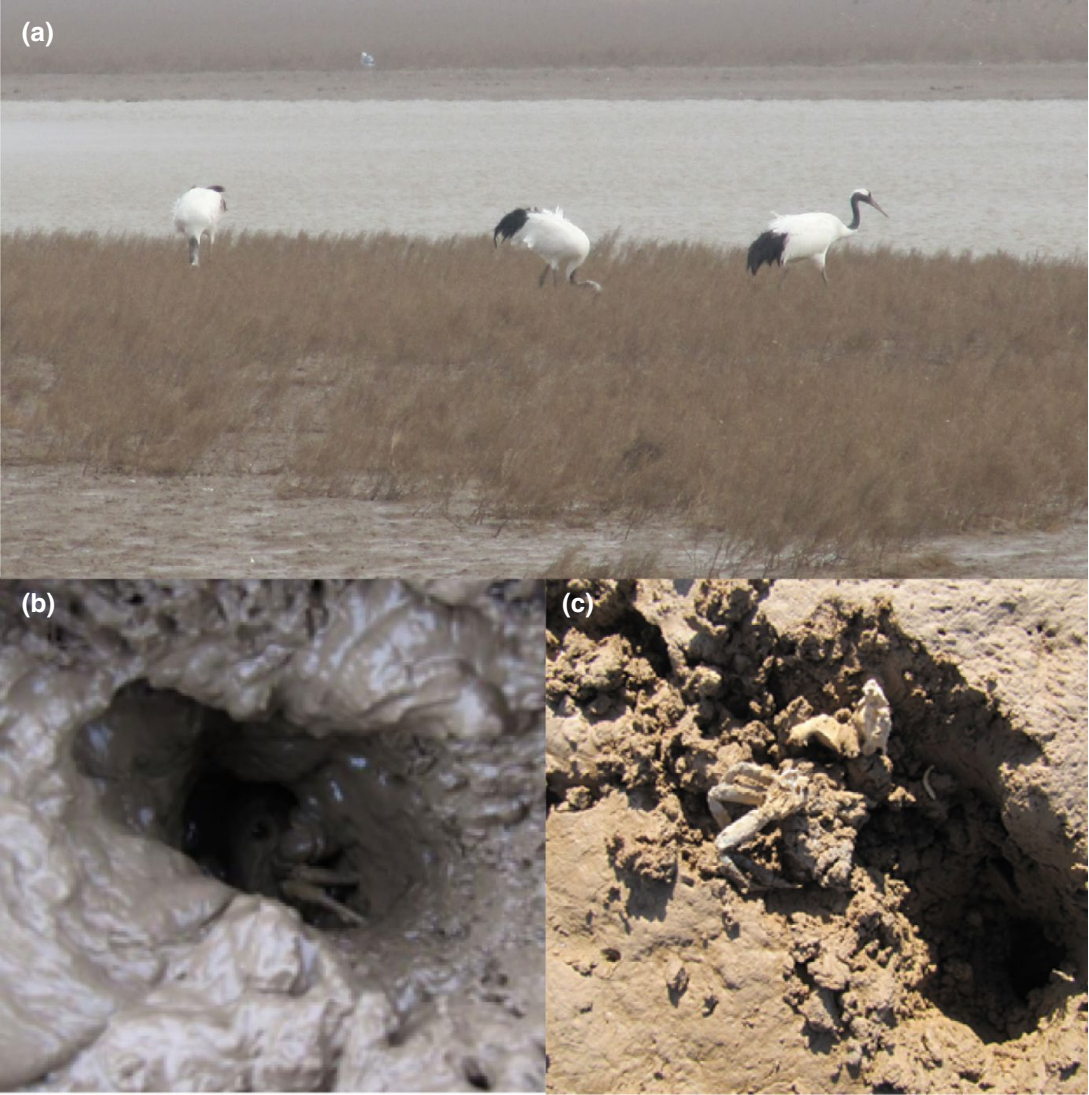
Showing Red‐crowned cranes foraging in *Suaeda salsa* saltmarsh (a), with mudflat crab presence at the burrow entrance (b) and remains of mudflat crabs found after recent foraging activity by Red‐crowned cranes (c)

The Red‐crowned crane (*Grus japonensis*) is a globally threatened migratory species (BirdLife International, 2016) that has undergone a severe population decline in China since 2000 (Su & Zou, 2012). Extensive loss and degradation of its primary breeding grounds in northeast China (Su & Zou, 2012) and extensive tidal land reclamation, wetland habitat invasion by smooth cordgrass (*Spartina alterniflora*), and expansion of oilfield production at wintering and staging areas (Cao, Xu, Le, Zhu, & Cao, 2015; Ma et al., 2009; Wang et al., 2019) have all contributed to its population decline. Red‐crowned cranes were formerly considered to be opportunistic feeders (Lee, Jablonski, & Higuchi, 2007; Ma, Wang, & Tang, 1999); however, at least one of our previous works (Li et al., 2014) revealed that mudflat crabs (*Helice tientsinensis*) in *Suaeda salsa* mudflat habitats form a crucial part of the species' winter diet in the Yellow River Delta. Li et al. (2014) also found that cranes increase their intake of mudflat crabs during late February to mid‐March, prior to their northward spring migration. This period coincides with increasing regional ambient temperature and the emergence of mudflat crabs from their winter hibernation burrows (Figure 1). Regional ambient temperature has been widely suspected to be a significant predictor of the timing of Red‐crowned crane autumn and spring migration. In addition, there are other large areas of similar *S. salsa* intertidal mudflats in two other crane stopover/wintering sites situated approximately 900km apart: the Liaohe River Delta (LRD) and Yancheng Nature Reserve (YNR) and mudflat crab populations occur at both these sites.

In this study, we use fecal analysis, observer‐based population counts, digital video recordings, and temperature data to determine whether increased ambient temperature affords better crab availability for Red‐crowned cranes during autumn and spring at three protected area stopover/wintering sites in eastern China. We predicted that: (1) mudflat crabs become more surface active with increasing burrow ambient temperatures; (2) crab surface activity would be limited to a temperature threshold; (3) crab activity would decline temporally during the crane's autumn migration period but increase during the spring migration period with increasing temperature; (4) crane diet will vary across different stopover/wintering sites and between autumn and spring; (5) cranes select and use staging/wintering sites with mudflat crabs when ambient temperature provides them with the opportunity to do so.

## 2 METHODS

### 2.1 Study sites

**FIGURE 2:**
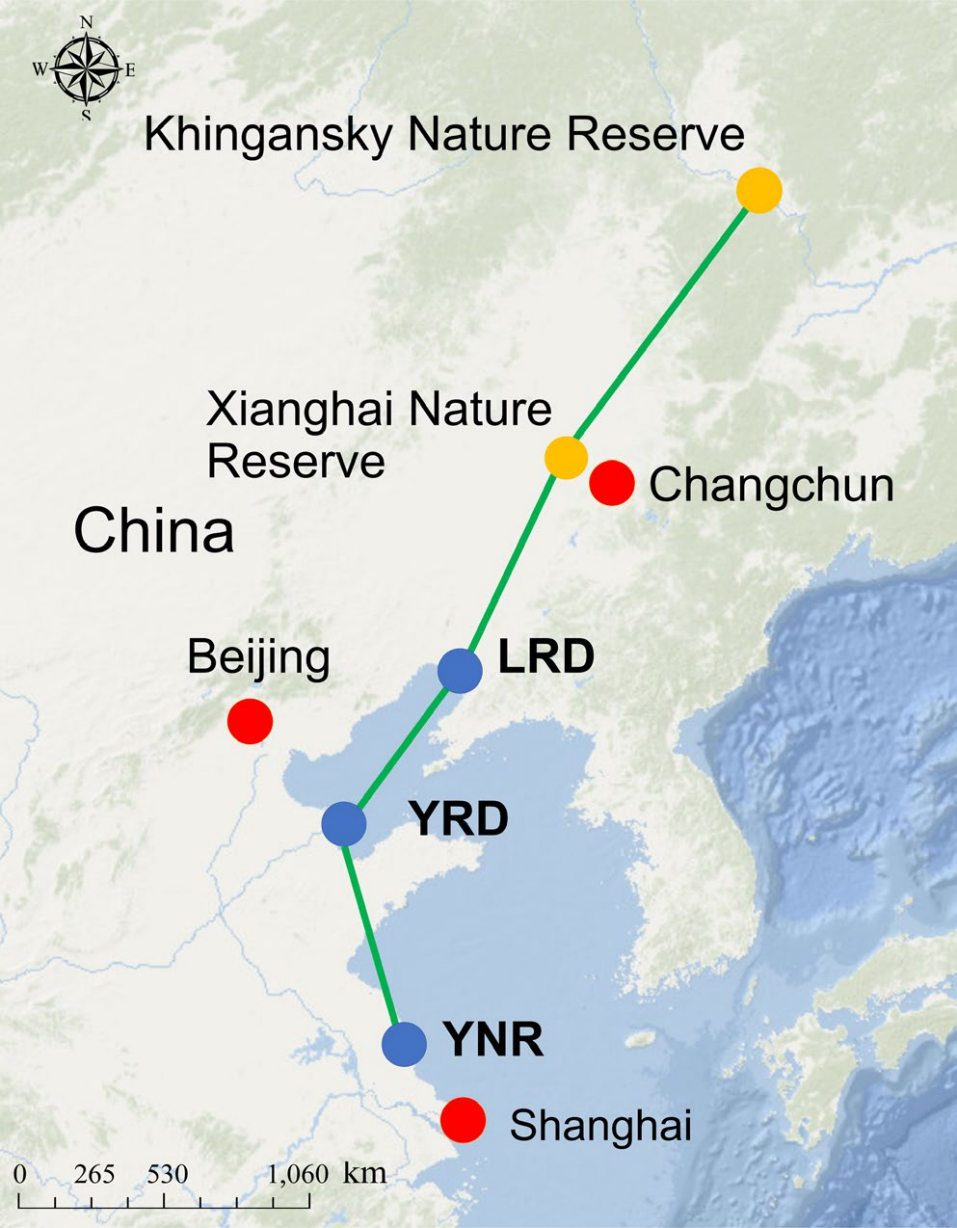
Location of three study sites (LRD: Liaohe River Delta Nature Reserve; YRD: Yellow River Delta Nature Reserve; YNR: Yancheng Nature Reserve) along the migratory route of Red‐crowned cranes in China (reference from Higuchi et al., 1998). Blue and orange circles depict the migratory/wintering sites and breeding sites, respectively, while red circles represent the proximity of three cities to the migratory flyway route of Red‐crowned cranes in China. The map was downloaded from IUCN Red List of Threatened Species (https://www.iucnredlist.org)

Fieldwork was conducted at three stopover/wintering sites used by the migratory Red‐crowned crane population along the eastern coast of China. From north to south, these sites are as follows: the Liaohe River Delta Nature Reserve (LRD: N 40.863°, E 121.755°), the Yellow River Delta Nature Reserve (YRD: N 37.802, E 119.139°), and the Yancheng Nature Reserve (YNR: N 33.592°, E 120.587°; Figure 2). Annual mean daily temperatures for LRD, YRD and YNR are 8.5°C, 11.9°C, and 14.2°C, respectively, with LRD and YRD separated by a distance of approximately 400 km, while YRD and YNR sites are approximately 600 km apart. Further details regarding the monthly mean and lowest temperature for each site are shown in Table S1. The weather conditions were typical for these sites during our survey periods. All three sites represent coastal wetland mosaics consisting of *S. salsa* saltmarshes, intertidal mudflats, reed marsh habitat, and artificial aquaculture fishponds (Li et al., 2011; Ma et al., 1999; Xia et al., 2017). In this region, the *S. salsa* saltmarsh—a preferred foraging habitat for migratory Red‐crowned cranes (Li et al., 2014)—has been greatly eroded in recent years due to a combination of land reclamation, changes to natural hydrological regimes, and invasion by non‐native smooth cordgrass. This latter threat is particularly prevalent in the southern YNR and also within the YRD, but to date, has not yet been recorded in the LRD (Wang et al., 2019).

### 2.2 Crab activity and ambient temperature data

The activities of mudflat crabs around their burrows were filmed using two digital cameras (Xiaoyi 4K, Shanghai, China). We randomly selected up to two crab burrows per day to film, with the camera positioned 0.5 m above the ground, to ensure an unobstructed view to the burrow entrance. Each recording lasted 8–9 hr from 8:00 a.m. to 16.00 hr or 17.00 hr. Recordings were made at YRD from November 7 to December 30 in 2014, and February 26 to March 28 in 2015, and at LRD from November 7 to November 29, 2014. No recordings were made during the spring of 2015 at LRD, because crabs were still in hibernation due to low ambient temperatures during Red‐crowned crane migration. In addition, no recordings were made at YNR because of limited logistical resources, but previous data have revealed that the Red‐crowned crane populations wintering there do feed on the crabs during the winter time (e.g., Ma et al., 1999). In total, we obtained 96 video samples of crab activity (LRD‐autumn: *n* = 12; YRD‐autumn: *n* = 36; YRD‐spring: *n* = 48).

Burrow ambient temperature was recorded at the entrance of each crab burrow at a depth of 2 cm using the Tinytag Plus 2 temperature data logger (TGP‐4520; Gemini Data Loggers, UK). We used one data logger per site, with LRD temperature sampling conducted during September 16 to December 24, 2014 (this logger failed to work during the following spring season) and YRD sampling conducted during November 20 to April 4, 2015. We selected one burrow for temperature data recording and did not move the data logger to a different burrow each day as we suspected there would be minimal variation in ambient temperature between different burrows. Crab activity at the burrow entrance (e.g., onset of activity and the percentage of activity time) was extracted from the video recordings using the Baofeng 5.0 digital player. Daily maximum and mean burrow ambient temperatures for each day were calculated to examine the influence of temperature on crab activity. Daily maximum ambient temperature data for the time period October 1, 2014, to April 30, 2015, for each site were downloaded from the website (http://www.tianqihoubao.com). These site‐related daily maximum ambient temperatures were used to explore the relationship between diet composition and migration timing of Red‐crowned cranes.

At YRD, we conducted additional monitoring of crab activity at burrow entrances. Every 2–3 days, we randomly selected a sampling area measuring 5 m × 10 m within the *S. salsa* habitat to conduct the experiment. Ten plots measuring 1 m × 1 m were randomly selected within the sample area. From each plot, we randomly selected 10 crab burrows and the location of which were marked with wooden poles. No sampling plot was repeatedly sampled, and the distance between plots was no less than 10 m. Before 9a.m. on each sampling day, we plugged the entrance of the burrows with mud from the immediate environment around the burrow entrance. We then checked each burrow 24 hr later to determine whether the burrow was open or closed and used these data as an indication of crab activity.

### 2.3 Fecal sample collection and prey identification

We collected a total of 902 fresh fecal samples of Red‐crowned cranes from their foraging or roosting sites between 2011 and 2015 (Table 1). Crane feces were easily distinguishable from that of other species by their large amorphous volume and always included large amounts of crab remains. On just a few occasions, we found Red‐crowned cranes feeding in the same habitat with Common crane (*Grus grus*) or Siberian crane (*Leucogeranus leucogeranus*), and only on these occasions we did not collect any fecal samples to avoid any error with allocating samples to the different crane species. We limited the number of fecal samples to ≤3 samples collected from each foraging or roost site to reduce the potential for pseudo‐replication. Only newly defecated samples were collected from the ground using a sterilized spoon, and these were subsequently stored in a sample tube and taken back to the field station (<8 hr travel time) and stored in refrigerator at −20°C before analysis.

**TABLE 1:** Percentage occurrence of different prey remains in Red‐crowned crane feces collected from three sites (YRD: Yellow river delta; LRD: Liaohe River Delta; YNR: Yancheng Nature Reserve) along the coastal of Yellow Sea between 2011 and 2015

Year	Site	Season	Number of feces samples (*n*)	Percent occurrence of prey remain (%)									
				Crab	Fish	Snail	Clam	Shrimp	Rice	Corn	Reed	Cattail	Others
2014	YRD	Spring	89	100.0	23.6	0.0	5.6	0.0	27.0	0.0	0.0	0.0	74.2
2015	YRD	Spring	367	99.5	13.1	11.7	8.4	0.0	0.0	0.0	0.3	0.0	17.7
2014	YRD	Autumn	95	100.0	9.5	4.2	2.1	0.0	0.0	0.0	0.0	0.0	31.6
2015	LRD	Spring	84	9.5	31.0	0.0	0.0	0.0	3.6	90.5	79.8	91.7	67.9
2014	LRD	Autumn	114	100.0	10.5	8.8	0.0	0.0	0.0	0.0	0.0	0.0	57.9
2011	YNR	winter	65	18.5	23.1	4.6	3.1	6.2	61.5	16.9	3.1	21.5	29.2
2014	YNR	winter	88	0.0	77.3	75.0	4.5	43.2	0.0	12.5	10.2	0.0	43.2
2011 and 2014	YNR	winter	153	7.8	54.2	45.1	3.9	27.5	26.1	14.4	7.2	9.2	37.3

Fecal analysis was conducted following the protocol of Li et al. (2014). Samples were disinfected by ultraviolet light for 30 min, then placed over a 0.3 mm sieve, and scoured under tap water for 10 min to separate soil and other matter. Indigestible parts were identified using a stereomicroscope, and food items were identified to the lowest possible taxonomic level, aided by comparisons with collected prey specimens. The percentage (%) of each type of prey remains for each sample and the percentage occurrence of prey remains in the total sample per season in each site were calculated to represent site and seasonal variations in crane diet composition.

### 2.4 Counts of migratory Red‐crowned crane

Red‐crowned crane population counts were conducted during the migration seasons in the YRD (November 1 to December 26, 2014; February 26 to March 26, 2015) and LRD (October 5 to November 30, 2014; March 10–11, 2015). Counts were conducted within the coastal *S. salsa* saltmarshes and adjacent intertidal mudflats known to be the main foraging habitat for Red‐crowned cranes (Li et al., 2017). We selected five vantage points at YRD and six vantage points at LRD, all were situated along the shoreline and from which it was easy to count all the individual cranes present in the coastal tidal flat. Each point was separated by a distance of approximately 2 km. Vantage points were visited in the same order for each count by two experienced observers (D.L. and J.Z.) spending 10 min counting cranes before moving to the next vantage point. Cranes were counted between 8:00 a.m. to 15.00 p.m. every 1–3 days and only during suitable weather conditions (i.e., no rain or strong winds) using telescopes (Swarovski ATS 80HD). Subsequently, we examined all count data from all points per day to exclude the possibility of double counting the same individuals from neighboring vantage points.

### 2.5 Statistical analyses

Crab activity datasets from all sites were pooled for the analyses without considering inter‐site differences. To test our first prediction, that mudflat crabs become more surface active with increasing burrow ambient temperature, we first used Pearson's correlations to examine the relationships between maximum daily and mean daily burrow ambient temperature with the onset (time) of crab activity time, and with the mean percentage of active crab burrows. In addition, we fitted two generalized linear models (GLMs) with the percentage time of crab activity at burrow entrances and percentage time crab activity outside of burrows on the mudflat as the response variables, with site (YRD, LRD), season (spring, autumn), daily maximum burrow ambient temperature and mean burrow ambient temperature included as predictor variables. Since both burrow ambient temperature response variables were positively correlated (*r* = .899, *p* < .001), we built these variables into the different models separately. We ran the GLMs with Poisson error structure and logit link function using the *glm* function included in the *MASS* package. We examined Wald test z scores to make inferences about each parameter estimate.

To test our second prediction (that crab surface activity would be limited to an ambient temperature threshold), we used a piecewise linear regression model to explore the relationships between percentage time of crab surface activity (combining the activity time both on the burrow entrance and outside the burrow on the mudflat) and maximum or mean burrow ambient temperature using the R package *segmented* (Muggeo, 2008). Differences in the burrow temperature at the onset of crab activity between different crane migration seasons were examined using independent two‐sample *t* tests. For our third prediction (mean percentage of active crab burrows would decline temporally during the crane's autumn migration period but increase during the spring migration period with increasing burrow ambient temperature), we fitted the data using Pearson's correlations. To test our fourth prediction (crane diet would vary across different stopover/wintering sites and between autumn and spring migration seasons), we examined seasonal site differences in the frequency of the presence of crab remains and the percentage of crab remains in crane fecal samples using chi‐square tests. We pooled all fecal sample data since we hypothesized that there was little variation in the foraging microhabitat of Red‐crowned cranes across the *S. salsa* wetland and that crab specialization was a function of ambient temperature. Finally, to test our fifth prediction, that cranes select and use staging/wintering sites with mudflat crabs when the site ambient temperature provides them with the opportunity to do so, we examine the percentage of crab remains in all fecal samples with daily maximum and daily mean site ambient temperatures with Pearson's correlations. All statistical analyses were conducted using R 3.6.0 (R Core Team, 2016), with significance set at 0.05, and the results expressed as mean ± standard error (*SE*).

## 3 RESULTS

1. Do mudflat crabs become more surface active with increasing burrow ambient temperature?

In total, 54% (*n* = 98) of the video samples of crab burrows revealed crab activity, either being recorded present on the entrance or walking outside of the burrow. Mean day time of the onset of crab activity was 11.41 ± 1.48 a.m. (*n* = 60, 24 hr) and varied from 09.16 am to 15.07 p.m. The onset of crab activity‐starting time was or very nearly negatively associated with the respective of daily maximum burrow ambient temperature (*r* = −.253, *p* = .051) and daily mean burrow ambient temperature (*r* = −.264, *p* = .041; Figure S1). Thus, mudflat crabs started to become active from their burrows earlier on days with warmer temperature. When all the burrow monitoring data were pooled, we found that the percentage of active crab burrows increased with increases in daily maximum burrow ambient temperature (*r* = .244, *p* = .193) and daily mean burrow ambient temperature (*r* = .296, *p* = .112) but these increases were not significantly correlated (Figure S2).

All GLMs, whether constructed using the daily maximum or daily mean burrow ambient temperatures, revealed significant positive influence of daily burrow ambient temperature on the mudflat crab activity either at burrow entrances or outside of burrows (Table 2). This suggests that mudflat crab activity was largely determined by burrow ambient temperature. In addition, the GLM models showed that there was significant variation in crab activity between seasons and sites, with crab activity being significantly higher in LRD than that in YRD. However, there was no significant difference in the percentage of time spent outside burrows between sites when daily maximum temperature was included in the model (Table 2).

1. Is crab surface activity limited to a temperature threshold?

**TABLE 2:** Parameter estimates (log‐odds) from GLMMs of daily temperature (maximum and mean) on crab activity

Responses	Variables	Estimate	Std. error	*z* Value	*p* Value
Percentage of time at burrow entrance	Intercept	**2.564**	**0.113**	**22.743**	<.001***
	Site (LRD)	**1.950**	**0.124**	**15.699**	<.001***
	Season (Autumn)	**−1.775**	**0.120**	**−14.836**	<.001***
	Daily maximum temperature	**0.016**	**0.006**	**2.502**	.00123**
Percentage of time outside burrows	Intercept	**−1.341**	**0.189**	**−7.102**	<.001***
	Site (LRD)	−0.018	0.091	−0.196	.8
	Season (Autumn)	**2.150**	**0.113**	**19.054**	<.001***
	Daily maximum temperature	**0.145**	**0.009**	**16.402**	<.001***
Percentage of time at burrow entrance	Intercept	**2.332**	**0.089**	**26.257**	<.001***
	Site (LRD)	**1.986**	**0.123**	**16.095**	<.001***
	Season (Autumn)	**−1.634**	**0.117**	**−13.930**	<.001***
	Daily mean temperature	**0.055**	**0.009**	**6.346**	<.001***
Percentage of time outside burrows	Intercept	**−0.779**	**0.161**	**−4.836**	<.001***
	Site (LRD)	**0.574**	**0.091**	**6.290**	<.001***
	Season (Autumn)	**1.947**	**0.102**	**19.003**	<.001***
	Daily mean temperature	**0.208**	**0.013**	**16.225**	<.001***

Note

Statistically significant estimates and standard errors (*SE*s) are highlighted in bold. The reference categories for “site” and “season” are “YRD” and “spring,” respectively. The temperature was recorded using a Tinytag Plus 2 temperature data logger (TGP‐4520; Gemini Data Loggers, UK).

**FIGURE 3:**
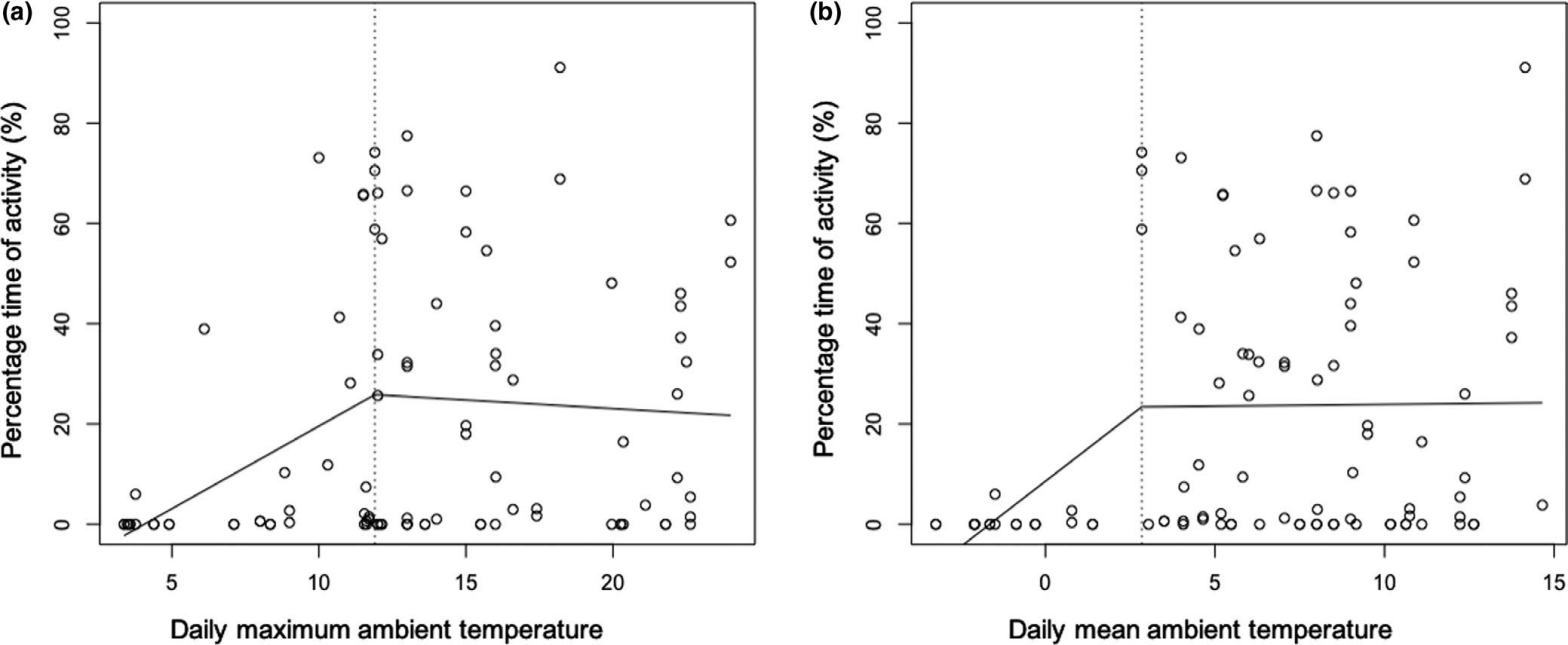
Relationships of crab activity and maximum daily or mean daily burrow ambient temperatures, with the thresholds identified by break points (a: 11.9 ± 2.2; b: 2.8 ± 2.4) estimated by piecewise linear regression models. The temperature was recorded using a Tinytag Plus 2 temperature data logger (TGP‐4520; Gemini Data Loggers, UK)

The average burrow ambient temperature for the onset of crab activity was 12.8°C ± 4.3 (25%~75% range, 10.37 ~ 15.38; *n* = 52), which was significantly lower in the autumn (11.1°C ± 4.2) than in spring (14.2°C ± 3.8; *t* = 2.799, *df* = 50, *p* = .007). The break point estimation of the piecewise linear regression shown that the percentage of time that crabs were recorded as being active at the burrow entrance and also outside of the burrows increased when daily mean ambient temperature was 2.8 ± 2.4°C and the daily maximum ambient temperature reached 11.9 ± 2.2°C (Figure 3; Table S2).

1. Does crab activity decline temporally during the crane's autumn migration period but increase during the spring migration?

**FIGURE 4:**
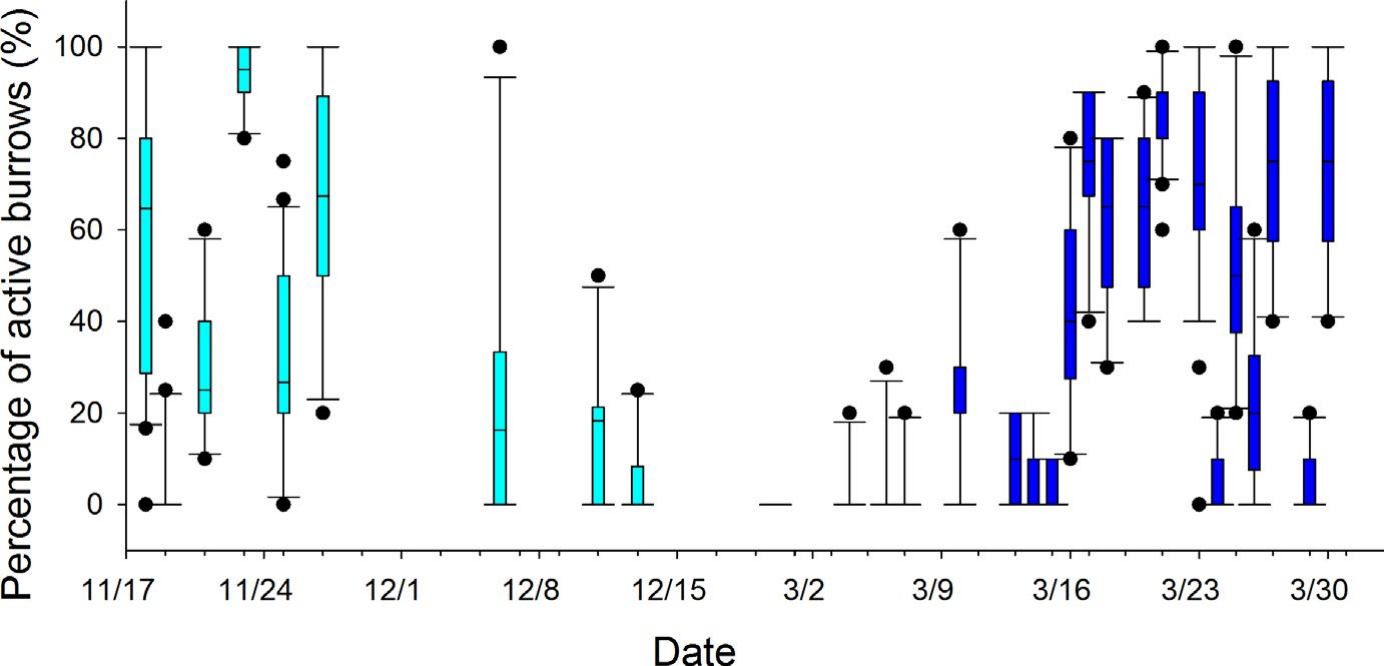
Temporal variation in the percentage of active crab burrows found at the YRD during the survey period. The light blue (left) and dark blue (right) boxes denote the samples from autumn and spring, respectively

We found a negative but nonsignificant relationship between mean percentage of active crab burrows and the date (*r* = −.562, *p* = .09, *n* = 10) in autumn (November 11–December 20) and a positive significant relationship between mean percentage of active crab burrows and date (*r* = .555, *p* = .011, *n* = 20) in spring (March 4–April 2) at YRD. This suggests that crab activity decreased from early December and increased again from early March in the following year (Figure 4).

1. Does crane diet vary across different staging/wintering sites and between autumn and spring?

**FIGURE 5:**
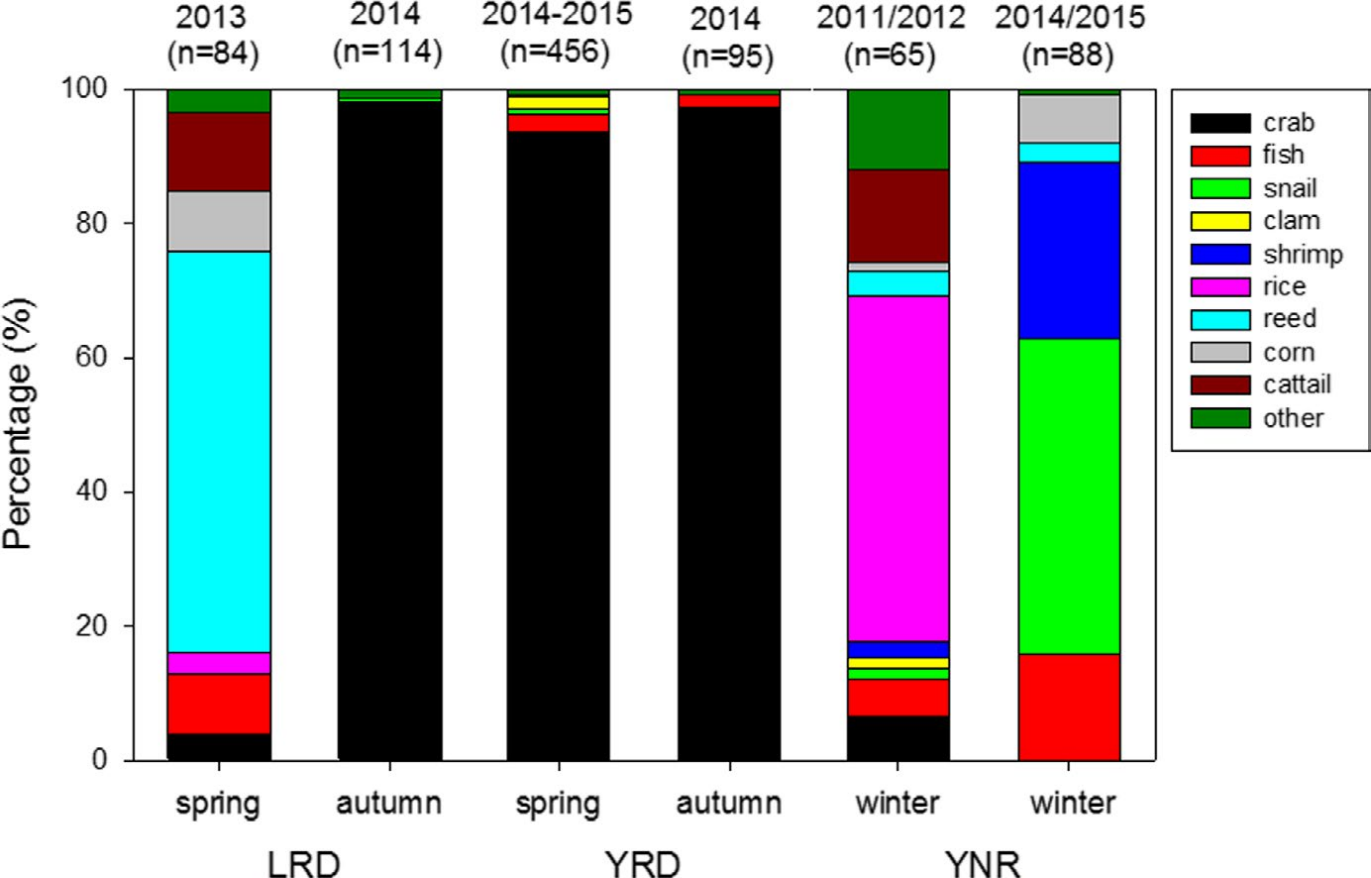
Temporal and site variations in the percentage of prey remains of Red‐crowned cranes during their annual migration and wintering in three important sites (north‐south: LRD: Liaohe River Delta Nature Reserve; YRD: Yellow River Delta Nature Reserve; YNR: Yancheng Nature Reserve) along the Yellow Sea

There were clear seasonal and site variations in the composition of prey in the crane's diet during the study period, with nine different categories of food remains identified in the fecal samples. There were significant differences in the frequency of the presence of crab remains between all three sites (chi‐square test: *χ* ^2^ = 420.18, *df* = 2, *p* < .001) and in the percentage of crab remains in crane fecal samples (*χ* ^2^ = 52.48, *df* = 2, *p* < .001). Crab remains were the dominant food type from crane feces collected in the YRD both in spring and autumn, and in the autumn of LRD (>90% in the percentage of prey remains), but not in the spring at LRD (*χ* ^2^ = 316.82, *df* = 3, *p* < .001; Figure 5). Three kinds of vegetation (corn, *Phragmites australis* shoots, and cattail *Typha orientalis* shoots) were found in fecal samples during the crane's spring staging stage at LRD (Figure 5 and Table 1) where crab remains only occurred in 9.5% of fecal samples and accounted for 3.93% ± 1.72 *SE* of all fecal content (*n* = 84). There was some variation in the diet composition of Red‐crowned cranes between the winter of 2011/2012 and 2014/2015 at YNR. At this site, crab remains only accounted for a small percentage (18.5%) of crane's fecal samples and less than 7% of the fecal contents in 2011/2012 winter, and no crab remains were found in crane feces in the YNR 2014/2015 samples. The dominant fecal remains were rice, snail, and shrimp in the 2011/2012 and 2014/2015 winters respectively at YNR (Figure 5). Fish were also a relatively important prey for Red‐crowned cranes, as it was recorded in samples from all three sites.

1. Do cranes select and use stopover and wintering sites with mudflat crabs when the site ambient temperature provides them with the opportunity to do so?

**FIGURE 6:**
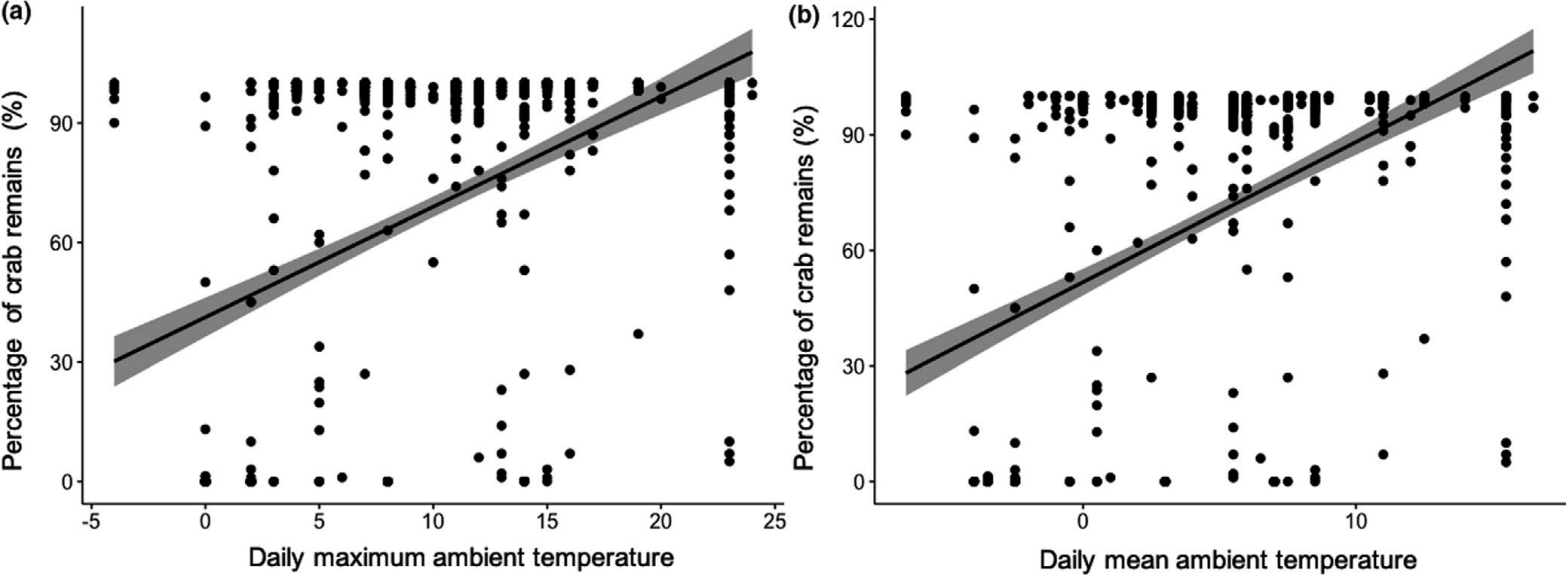
Relationships between crab remains in the feces of Red‐crowned cranes with daily maximum ambient temperature (a: °C) and daily mean ambient temperature (b: °C). The data of temperature were downloaded from website (http://www.tianqihoubao.com)

**FIGURE 7:**
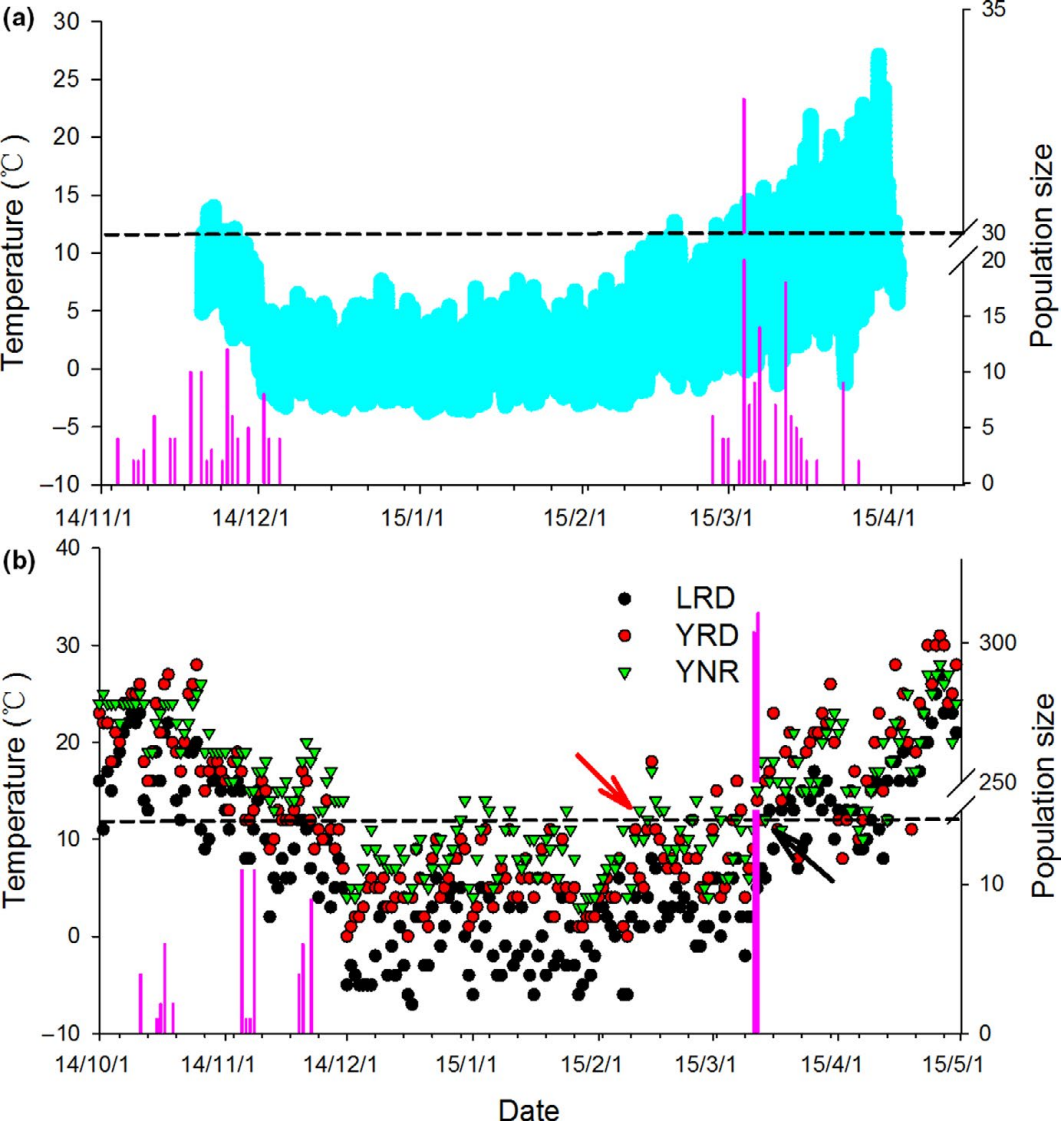
Relationship between the use of three staging/wintering sites by Red‐crowned cranes and the site‐specific variation in ambient temperature (LRD: Liaohe River delta; YRD: Yellow River Delta; YNR: Yancheng Nature Reserve). a denotes ambient temperature recorded using a Tinytag Plus 2 temperature data logger (TGP‐4520; Gemini Data Loggers, UK) in the YRD; b denotes site variation in the daily maximum ambient temperature (Download: http://www.tianqihoubao.com). The dotted line represented the threshold value of crab activity temperature identified using piecewise linear regression models. The number of individual cranes in b represents the migratory pattern of cranes in Liaohe River Delta (LRD). The red and black colored arrows represent the date of the daily maximum temperature exceeding the threshold value of 11.9°C in the YRD (February10, 2015) and LRD (March 15, 2015)

When all the fecal samples were pooled, there was a significant positive correlation between the percentage of crab remains in crane feces with both daily maximum site ambient temperature (*r* = .42, *p* < .001) and daily mean site ambient temperature (*r* = .46, *p* < .001; Figure 6). Red‐crowned cranes began arriving at YRD on their autumn migration from mid‐October and the majority of cranes had left the area by early December, although some individuals wintered there (see also figure 2 in Li et al., 2014). The stopover time of southward migratory cranes at YRD overlapped with a noticeable decline in the daily maximum ambient temperature to less than 11.9°C (Figure 7a). In spring, Red‐crowned cranes arrived at YRD during mid‐ February to early March, coinciding with the daily maximum ambient temperature increasing to 11.9°C (Figure 7a). Red‐crowned cranes began staging at LRD from early October to the end of November, with numbers peaking in early November when local daily maximum ambient temperatures reached above 11.9°C (Figure 7b). In spring, cranes arrived in early March, before daily maximum ambient temperatures increased toward 11.9°C (Figure 7b), and consequently mudflat crabs were not available to cranes during this migration stage. The wintering stage of Red‐crowned crane in the YNR was from November to early February, when the daily maximum ambient temperature was higher than 11.9°C, and crab remains were found in the diet of cranes wintering in this area.

## 4 DISCUSSION

In this study, we have shown how variation in burrow ambient temperature influences diet composition of the migratory Red‐crowned crane population across three wintering/stopover sites and different migration seasons through its influence on crab prey availability. Mudflat crab activity was largely influenced by burrow ambient temperature (prediction 1) and crab activity significantly increased at a burrow ambient temperature threshold of 12 ~ 13°C (prediction 2). Crab activity tended to advance with warmer ambient temperature, with the percentage of active burrows declining in the autumn and increasing again during the spring migration (prediction 3). Temperature‐related crab availability also has a significant influence on variation in the amount of crab prey found in crane feces between different staging/wintering sites and migration seasons (prediction 4). Finally, we also found a significant, positive relationship between the percentage of crab remains in the crane feces and local daily ambient temperature, and a degree of congruence between local ambient temperature and the arrival and departure timing of Red‐crowned cranes at our three sites. This suggests that temperature‐depended mudflat crab activity may provide an important time window for migratory Red‐crowned cranes (prediction 5).

Red‐crowned cranes typically spend 4–5 months staging for winter in the Yellow Sea coastal region (Higuchi et al., 1998; Ma et al., 1999; Su & Zou, 2012). To maintain their daily energy requirements, cranes should rely on predictable high‐energy prey resources at these sites. Similar patterns of temporal synchrony in migration timing and prey availability have been documented for several migratory shorebird species. For example, the seasonal spawning of horseshoe crab (*Limulus polyphemus*) at Delaware Bay, USA, provides a critical nutrient‐rich dietary resource (eggs) for migratory populations of red knot *Calidris canutus*, ruddy turnstone *Arenaria interpres*, sanderling *C. alba*, and semipalmated sandpiper *C. pusilla* (Castro & Myers, 1993; Tsipoura & Burger, 1999). Increasing activity of two crab species *Panopeus africanus* and *Callinectes marginatus*, during late spring is critical for migrating whimbrel populations (*Numenius phaeopus*) for their spring department from Banc d'Arguin, Mauritania (Zwarts, 1990). Previous studies suggested that Red‐crowned cranes were opportunistic feeders that consume primarily fish, amphibians, invertebrates, shrimps, and plant matter (Lee et al., 2007; Ma et al., 1999; Zou et al., 2016). However, our results and that from our previous work (Li et al., 2014) confirm that at a small number of their critical wintering sites, Red‐crowned cranes are largely dependent on mudflat crabs. Among other bird species, curlews (Piersma, 1986; Zharikov & Skilleter, 2002) and some gull species (Beron et al., 2011; Ellis et al., 2005) are considered as crab‐eating specialists. Whooping crane (*Grus americana*) is the only other crane species currently known to depend on crabs (Hunt & Slack, 1989) and whose winter mortality rates are correlated with declines in populations of blue crab (*Callinectes sapidus*) (Pugesek, Baldwin, & Stehn, 2013).

Although mudflat crabs were the dominant prey of Red‐crowned cranes staging in the LRD during the autumn, there were almost no crab remains found in crane feces at this site during the spring migration. The absence of mudflat crabs in the crane's spring diet at LRD may be largely due to the lower burrow ambient temperature at LRD on their arrival that restricted crab activity/availability (Figure 7b). This also highlights the trade‐off made by migratory cranes—trying to synchronize the timing of their migration to exploit a suitable prey resource, with the need to reach their breeding ground. Ideally, Red‐crowned cranes should time their northward spring migration to arrive at LRD some 3–4 weeks later than currently documented, when ambient temperatures are sufficiently high to influence greater crab activity. Our data do reveal that cranes staging at LRD may be able to adapt to a degree to feed on other food resources during the spring, and elsewhere, there are some reports that wintering Red‐crowned cranes utilize anthropogenic habitats such as rice field (Li et al., 2013; Ma et al., 1999; Wang, Li, Beauchampe, & Jiang, 2011). Studies have revealed that numerous wading bird species vary their prey choice in response to variation in the availability of their different prey species (Zwarts & Wanink, 1993). Whether crane use of anthropogenic habitats is linked to burrow ambient temperature and crab availability remains unknown. Further research is needed to determine the degree with which these and other food types, in particular, corn, which is used as a supplementary feeding strategy by the protected area management at YNR and LRD, can nutritionally compensate for the lack of crab prey in their diet during pre‐migration (e.g., daily energy requirements, fat accumulation).

The prevalence of mudflat crabs in the crane's diet might be related to the ease of catching slower moving individuals during days with ambient temperatures at around the threshold of 12 ~ 13°C, or by catching inactive individuals found warming themselves at burrow entrances. Research from NW Europe has shown that invertebrate prey availability for visually feeding wading birds is significantly reduced when sediment surface temperatures fall below 3–6°C and that fewer prey are available for migrant species at sites with much colder ambient temperature (Zwarts & Wanink, 1993). We lacked detailed data to determine whether cranes resort to feeding largely on crabs within their burrows or at burrow entrances either during colder days, or on days when ambient temperature reach above the threshold (as opposed to foraging more on surface active crabs). Little is known about anti‐predator behavior of mudflat crabs during crane migration seasons at our study sites, and further research is needed to identify the period when mudflat crabs are most vulnerable to predation by cranes and whether any such periods correlate with spatial patterns of habitat use (burrow vs. mudflat surface).

The migratory timing of birds is multi‐mechanistic, determined by numerous biological, ecological, and environmental factors (e.g.,Haest, Hüppop, van de Pol, & Bairlein, 2019; Hedenström, Alerstam, Green, & Gudmundsson, 2002; Newton, 2008). Temperature‐related prey availability for wading birds has long been recognized but their influence on the migratory patterns of birds has only recently been addressed (Brisson‐Curadeau, Elliott, & Côté, 2020; Nebel & Thompson, 2005; Therrien et al., 2017). Although we have found some congruence between the migration timing of Red‐crowned cranes at three staging/wintering sites with the temperature‐dependent temporal availability of mudflat crabs, the arrival of cranes at these staging/wintering sites will be influenced by other factors. To our knowledge, this study provides the first evidence to show the influence of burrow ambient temperature on crab activity/availability for migratory Red‐crowned cranes, and the influence this has on crane diet across different coastal protected areas. These findings are significant for addressing the conservation of mudflat crabs and *S. salsa* habitat in the Yellow Sea region, which are threatened by anthropogenic land‐use and exotic *Spartina alterniflora* invasion (Wang et al., 2019; Li *et al*., unpublished data). Clearly, both representing critical resources for migratory Red‐crowned cranes and identifying links between prey availability and migration timing across multiple stopover sites would provide more comprehensive data for improved migratory bird habitat conservation management in the region. Since mudflat crab represents a temperature‐related food resource, it will be critical to determine whether Red‐crowned cranes wintering in coastal China can adjust their migration timing to response to further variation in weather and/or phenology along their migration routes due to the predicted impacts of global climate change. Further data from their breeding ground regarding arrival times, territory establishment, and breeding success are needed to shed more light on these trade‐offs.

## CONFLICT OF INTEREST

None declared.

## AUTHOR CONTRIBUTION

**Donglai Li:** Conceptualization (equal); Data curation (equal); Formal analysis (equal); Funding acquisition (equal); Investigation (equal); Methodology (equal); Project administration (equal); Resources (equal); Software (equal); Supervision (equal); Validation (equal); Visualization (equal); Writing‐original draft (equal); Writing‐review & editing (equal). **Jing Zhang:** Investigation (equal); Methodology (equal); Validation (equal). **Lingyu Chen:** Investigation (equal); Methodology (equal); Validation (equal). **Huw Lloyd:** Formal analysis (equal); Software (equal); Validation (equal); Visualization (equal); Writing‐review & editing (equal). **Zhengwang Zhang:** Conceptualization (equal); Funding acquisition (equal); Project administration (equal); Validation; Writing‐review & editing.

## Supporting information

Figure S1

Figure S2

Tables S1‐S2

## Data Availability

Study data are publicly available in Dyrad Digital Repository (https://doi.org/10.5061/dryad.s1rn8pk5k).
