# Supplementary figures and images for: Burrow ambient temperature influences Helice crab activity and availability for migratory Red‐crowned cranes Grus japonensis
Source: Ecol Evol. 2020 Sep 18;10(20):11523–34. doi: 10.1002/ece3.6788 (PMC7593175; doi:10.1002/ece3.6788)

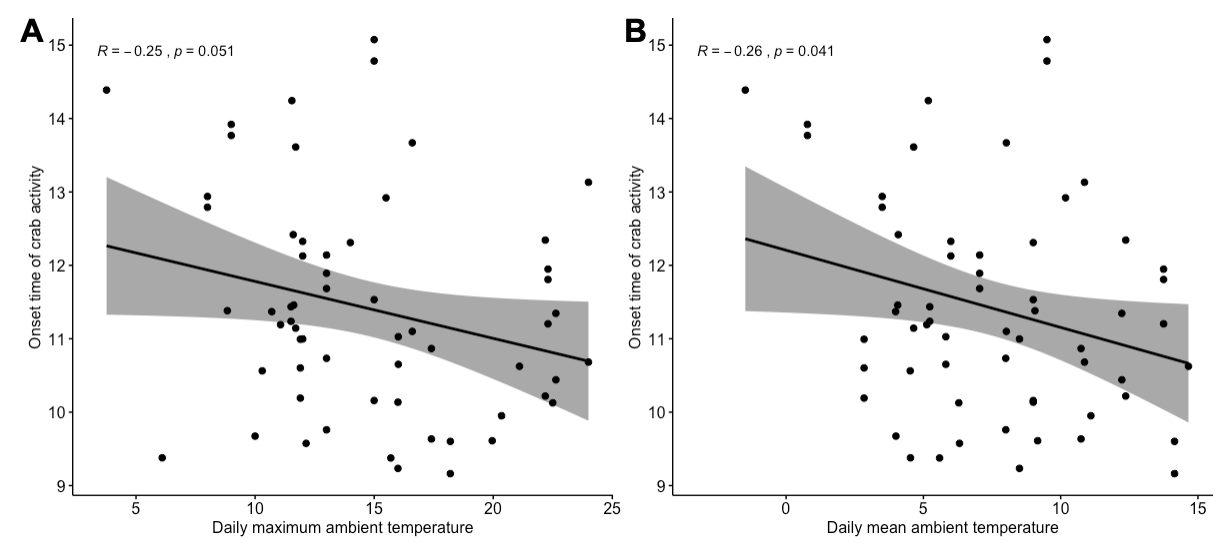

Supplement: Supplementary file 1 — Figure S1 [file ECE3-10-11523-s001.tif]

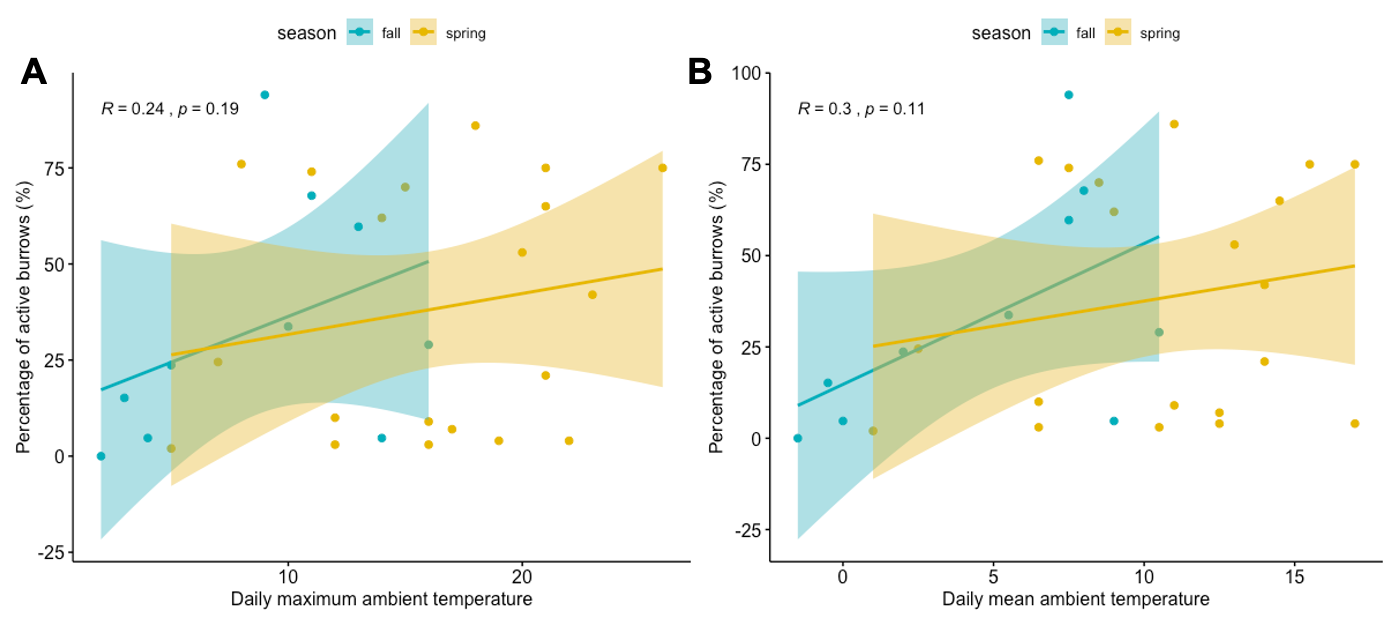

Supplement: Supplementary file 2 — Figure S2 [file ECE3-10-11523-s002.tif]
