# Supplementary material for: Burrow ambient temperature influences Helice crab activity and availability for migratory Red‐crowned cranes Grus japonensis
Source: Ecol Evol. 2020 Sep 18;10(20):11523–34. doi: 10.1002/ece3.6788 (PMC7593175; doi:10.1002/ece3.6788)
Supplement: Supplementary file 3 — Tables S1‐S2 [file ECE3-10-11523-s003.docx]

Table S1.Site variations in the mean daily temperature and lowest temperature for each month covering the migratory and wintering time of Red-crowned Cranes at three study sites (LRD: Liaohe River Delta Nature Reserve; YRD: Yellow River Delta Nature Reserve; YNR: Yancheng Nature Reserve). The table was summarized of the data from October 2014 to March 2015 downloaded from website (http://www.tianqihoubao.com)

| **Temperature** | **Site** | **October** | **November** | **December** | **January** | **February** | **March** |
| --- | --- | --- | --- | --- | --- | --- | --- |
| Mean daily temperature | LRD | 12.3 | 4.0 | -5.4 | -6.2 | -2.7 | 3.8 |
|  | YRD | 16.5 | 8.3 | 0.6 | 1.1 | 2.6 | 9.2 |
|  | YNR | 18.1 | 11.7 | 3.2 | 4.1 | 5.1 | 9.6 |
| Lowest temperature | LRD | -3.0 | -7.0 | -16.0 | -16.0 | -16.0 | -10.0 |
|  | YRD | 6.0 | -4.0 | -6.0 | -7.0 | -7.0 | -5.0 |
|  | YNR | 7.0 | 2.0 | -5.0 | -4.0 | -1.0 | 0.0 |

Table S2. Estimated coefficients and break-points fitted by the piecewise linear regression model with segmented relationships between percentage of time of crab surface activity and daily maximum or mean burrow ambient temperature. The temperature was recorded using a Tinytag Plus 2 temperature data logger (TGP-4520; Gemini Data Loggers, UK).

| Variables | Estimate | SE | t value | P |
| --- | --- | --- | --- | --- |
| (Intercept) | -13.38 | 10.86 | -1.23 | 0.22 |
| Daily maximum burrow ambient temperature (slope 1) | 3.30 | 1.24 | 2.66 | 0.01 |
| Daily maximum burrow ambient temperature (slope 2) | -0.34 | 0.84 | -0.41 | NA |
| Estimated Break-Points | **11.90** | **2.24** |  |  |
| (Intercept) | 8.59 | 5.59 | 1.54 | 0.13 |
| Daily mean burrow ambient temperature (slope 1) | 5.22 | 2.88 | 1.81 | 0.07 |
| Daily mean burrow ambient temperature (slope 2) | 0.07 | 0.93 | 0.07 | NA |
| Estimated Break-Points | **2.84** | **2.42** |  |  |
